# Positive and instructive anti-smoking messages speak to older smokers: a focus group study

**DOI:** 10.1186/s12971-015-0027-x

**Published:** 2015-01-24

**Authors:** Janine K Cataldo, Mary Hunter, Anne Berit Petersen, Nicolas Sheon

**Affiliations:** Department of Physiological Nursing, University of California San Francisco, Parnassus Avenue, San Francisco, CA USA; Department of Medicine, University of California San Francisco, 550 16th St, 3rd Floor, San Francisco, CA USA

**Keywords:** Older smokers, Tobacco, Smoking, Tobacco warnings

## Abstract

**Background:**

Smokers over the age of 45 are the only group with an increase in smoking prevalence, are the least likely to quit smoking, and bear most of the burden of tobacco-related disease. Research characterizing older adult perceptions of warning labels and anti-tobacco messages has not been reported in the literature. The purpose of this study was to describe whether older smokers perceived warning labels and anti-tobacco messages as effective for the promotion of smoking cessation. A secondary aim was to explore what types of messages and message delivery formats are most relevant to older adult smokers.

**Methods:**

This focus group study is part of a larger study to characterize older smokers’ perceptions of the risks and benefits associated with conventional and emerging tobacco products and determine the extent to which these perceptions relate to exposure to pro- and anti-tobacco messages. From April 2013 to August 2014 we conducted eight focus groups with 51 current and former smokers a focus group study in urban and suburban California. A semi-structured format about current use of conventional and emerging tobacco products was used. Participants were asked to recall and comment on examples of warning labels and anti-tobacco messages. Data were transcribed and thematically coded.

**Results:**

Warning labels and anti-smoking messages were seen as ineffective for smoking cessation motivation among older California smokers. Positive framed anti-tobacco messages were identified as most effective. Text-only warnings were seen as ineffective due to desensitizing effects of repeated exposure. Negative messages were described as easy to ignore, and some trigger urges to smoke. Older adults are knowledgeable about the risks and health effects of smoking. However, they tend to be less knowledgeable about the benefits of cessation and may underestimate their ability to quit.

**Conclusion:**

These findings suggest that messages with a positive frame that outline immediate and long-term benefits of cessation would be an effective approach for long-term smokers.

Current anti-tobacco messaging was generally not seen as effective for smoking cessation among long-term smokers.

**Electronic supplementary material:**

The online version of this article (doi:10.1186/s12971-015-0027-x) contains supplementary material, which is available to authorized users.

In the US, Cigarette smoking is responsible for more than 480,000 annual deaths. In 2012, the overall smoking prevalence was 18.1% but for the 45–64 year old group it was higher, at 19.5%. Among adults over 65 it was lowest of all groups at 8.9% [1]. This is most likely due to the fact that many smokers have succeeded in quitting by the age of 65 [2] and life expectancy for smokers is at least 10 years shorter than for non-smokers [1].

Over the last 50 years, tobacco control efforts in the US have been youth oriented and have significantly decreased smoking prevalence. Between 1965 and 1994, the proportion of smokers in the US under the age of 65 decreased by 18.4%. In contrast, smoking prevalence in the over 65 age group declined only 5.9% [3]. From 2005 to 2012, smoking prevalence for all ages combined declined from 20.9% to 18.1%, and significant reductions in smoking prevalence occurred among the 18–24 age group (i.e., 24.4% to 17.3%). However, during this same time, the over 65 age group was the only group with an increase in smoking prevalence (8.9% to 10.6% for men and 8.6% to 8.9% overall) [4]. By 2050, the over 65 age group in the US will double [3]. The costs of smoking are significant; in 2012, Californians paid about $6.5 billion toward adult tobacco-related health care costs, more than $400 per taxpayer [5]. If these trends remain constant, the number of older smokers and the cost of smoking-related disease will double [6]. Research characterizing older adult (>45 years) perceptions of warning labels and anti-tobacco messages has not been reported in the literature.

## Background

### Older adult smokers

Smokers over the age of 45 bear most of the burden of tobacco-related disease [7]. Older smokers frequently face economic and social disadvantages yet are often ignored in discussions of marginalized populations impacted by tobacco [8-10]. Older smokers are least likely to quit of any age group, and are least likely to appreciate the benefits of cessation [7,11,12]. This may be due in part to tobacco companies’ use of marketing to reduce perceptions of harm associated with tobacco use, increase perceptions of the social acceptability of smoking, and ultimately encourage tobacco use [13-15]. Tobacco industry marketing exposure distorts perceptions about the availability, use, and risks of tobacco [16].

### Warning labels

In the US, health warnings on cigarette packages have been required since 1965. These messages consist of small font text boxes such as “Warning: The Surgeon General Has Determined that Cigarette Smoking is Dangerous to Your Health”. Efforts are ongoing to adopt the larger graphic warnings used in other countries that have been found to be effective with respect to perceptions of risk and cessation-related behaviors [17].

Warning labels on cigarette packages have been found to inform smokers about the health hazards of smoking and encourage smokers to quit; they are an ideal way to communicate with smokers because the intervention is delivered at the time of smoking. Two-thirds of all smokers indicate that the package is an important source of health information and is strongly associated with an intention to quit smoking [18] and is effective in discouraging initiation and encouraging cessation [19-21]. Evidence indicates that graphic warnings are more effective than text-only messages, induce a greater emotional response, are more likely to retain their salience over time, and increase awareness of health risk [22,23].

Repeated exposure renders cigarette package warning labels less effective as smokers become inured to negative messages over time [24-26]. A pack-a-day smoker could be exposed to the warnings more than 7,000 times a year [23]. Bala and colleagues (2008) conducted a meta-analysis on mass media campaigns but the sample was small (i.e. 11 studies) and adult was defined as >20 years old effectiveness was measured by smoking behavior [27]. Durkin et al. [28] concluded based on Bala’s work that campaign effectiveness does not consistently differ by gender and age [28]. However, this does not speak to the effectiveness of the messages or type of messages; further research is needed to determine the type and style of messages needed to reach older adults.

### Anti-tobacco messages

Since 1990, the California Tobacco Control Program (CTCP) has been producing hard-hitting educational ads that have contributed to a significant decrease in the number of Californians who smoke by changing the social acceptance of tobacco use. The many foci of these campaigns have included messages to: reduce secondhand smoke exposure; counter the tobacco industry’s deceptive marketing efforts to initiate new users; motivate and assist tobacco users to quit; and educate about the harmful effects of toxic tobacco waste on the environment. The CTCP Media Campaign includes television, radio, print, billboard ads, and online efforts in several languages (http://www.tobaccofreeca.com/ads/about/).

Evidence indicates that mass media can promote quitting and reduce adult smoking prevalence. When different message types were compared, one study found messages about negative health effects were the most effective at generating increased knowledge, beliefs, positive perceived effectiveness ratings, and quitting behaviors [28]. A few studies further suggest that negative health effect messages may also contribute to reduction in socioeconomic disparities in smoking [28]. However, research with older adults, although limited, has shown that positive health messages that emphasize benefits are more effective than messages that emphasize negative consequences [29]. Furthermore, there are research findings that indicate older adults remember the content in positive health messages better than the content in negative messages [30]. Numerous focus group studies with older adults have reported a preference for positive health warning messages over fear-based appeals and graphic warnings [23,31]. Studies that specifically address older adults perceptions of the type and impact of anti-tobacco messages are needed. The aims for this focus group study were to: 1) describe whether older smokers perceived warning labels and anti-tobacco messages as effective for the promotion of smoking cessation and 2) explore what types of messages and message delivery formats were most relevant to older adult smokers.

## Methods

The institutional review board at the University of California San Francisco approved this study. For this study, participants met in eight 90-minute focus groups to discuss topics related to cigarettes and alternative tobacco products. This focus group study is part of a larger study to characterize older smokers’ perceptions of the risks and benefits associated with conventional and emerging tobacco products and determine the extent to which these perceptions relate to exposure to pro- and anti-tobacco messages. To explore perceived effectiveness of warning messages, participants were asked to recall and describe anti-tobacco messages and were asked questions about their perceived effectiveness (e.g. Would/did this influence your smoking behavior? Do you think this is an effective anti-smoking message? If not, what would work for you?)

### Data collection

**Figure 1:**
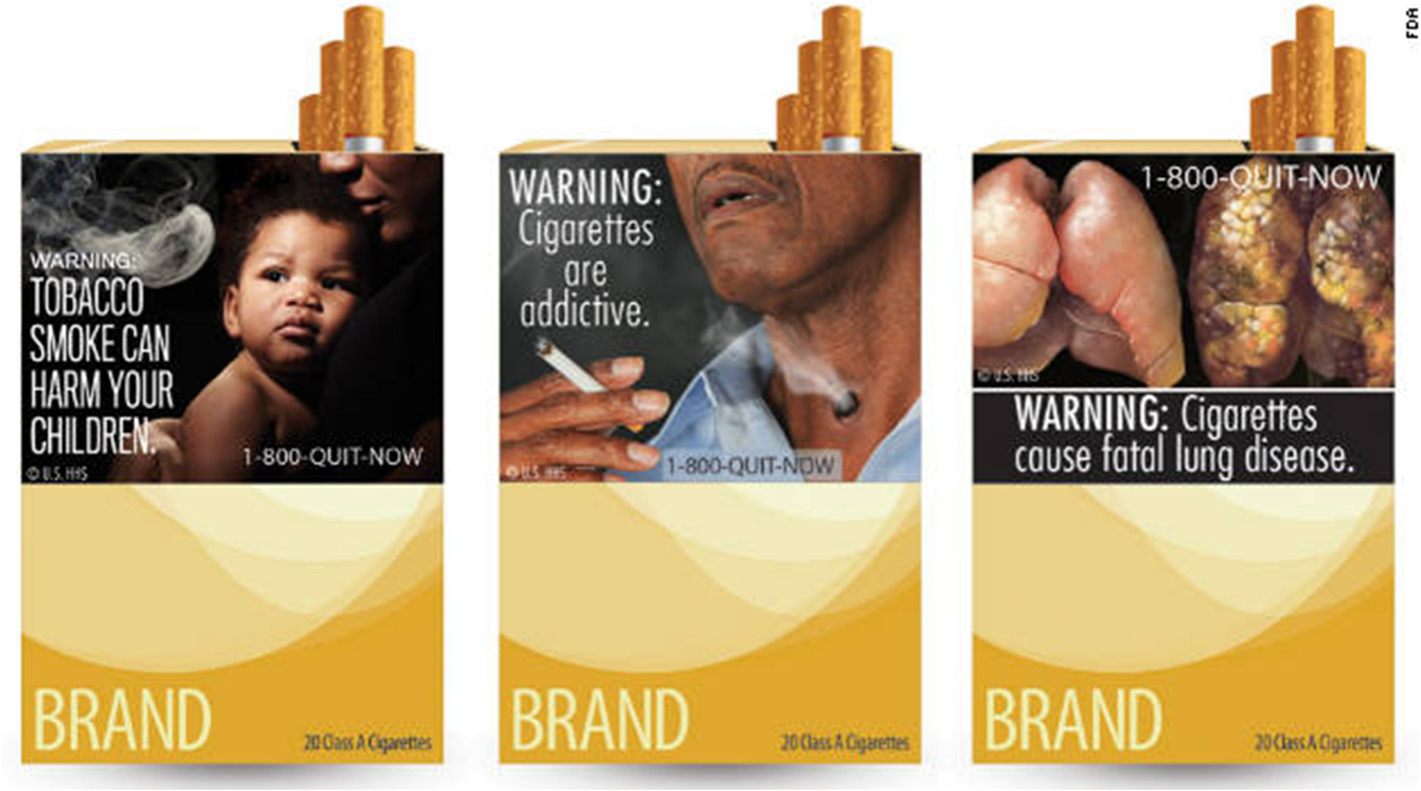
**Proposed FDA warning labels.**

Participants were recruited from urban and suburban communities in California using flyers and online classified advertisements. Interested participants called the research number included in the advertisements and they were asked pertinent inclusion criteria questions. Inclusion criteria included age >45 years old (no upper age limit), current smoker or former smoker (smoker defined as “has smoked at least 100 cigarettes in a lifetime” and former smoker having quit in the last 5 years), able to speak and read English. If the participant qualified they were provided with information about the time and place of the focus group, they were asked for a contact phone number or email and they were contacted the day before the group to confirm their participation. At the group site, participants provided informed written consent and completed a demographic questionnaire that included tobacco use history. The focus groups followed a semi-structured format with open-ended questions that permitted exploration of tobacco-related topics as they emerged in the discussion. They were initially asked to discuss their current tobacco use, current interest in quitting, and the places and situations in which they avoid smoking or use alternative tobacco products. Participants were asked to comment on their exposure to anti-smoking messages. They were asked to recall and comment on cigarette package warning labels and comment on warning labels a handout with pictures of the 2012 proposed FDA warning labels was provided. (see Figure 1) (http://www.fda.gov/TobaccoProducts/Labeling/ucm2024177.htm).

### Data analysis

The focus groups were transcribed by a professional transcriptionist who was instructed to label each speaker turn as a separate paragraph. Within Microsoft Word, we used the “convert text to table function” to convert the transcripts into a continuous vertical table of cells with each cell containing one speaker turn. We then pasted these tables into a Microsoft Excel spreadsheet, adding columns identifying the date and location of the focus group, and numbering each speaker turn, or cell row. Each speaker turn thus provided a unit of analysis for the application of codes varying in size from a single word to several paragraphs. To code these segments we created a column for each code, and indicated the presence of the code by adding the coder’s initials to the intersection of the segment row and the code column. Coding was completed for the larger study and included codes related to anti-tobacco messages.

This spreadsheet enabled us to easily view the data segments and the codes applied to them in analytically useful ways. First, the transcripts were read in their original, sequential context. The segments were indexed into topical codes enabling us to see horizontal patterns in the co-occurrence of topics for each data segment. An initial 53 topical codes were identified from the transcripts and through discussions among the three coders the code list was subsequently reduced iteratively to 42 as we combined topical codes with significant overlap (Table 1). By using their initials when coding segments, coders were able to identify and discuss discrepancies in how each coder applied codes to each data segment until we reached agreement.

**Table 1:** **Topical codes**

**Code category**	**Code**	**Speaker turns coded**
Cessation	Doctor Interaction	88
Cessation	Successful Quit Attempt	63
Cessation	Other Quit Methods	47
Cessation	Unsuccessful Quit Attempt	43
Cessation	Patch	28
Cessation	Chantix	21
Cessation	Fatalism	8
Cessation	Not Currently Smoking	7
Cessation	Wellbutrin	6
Consequences	Health Effects	110
Consequences	Lung Cancer	75
Consequences	Addiction	67
Consequences	Smell Bothersome	63
Consequences	Second Hand Smoke	29
Consequences	Cosmetic Effects	3
Contexts	Nostalgia	147
Contexts	Sex	50
Contexts	Pot	48
Contexts	Movies/Tv	47
Contexts	Like About Smoking	32
Contexts	Social Triggers	32
Contexts	Sport	28
Contexts	Alcohol	21
Contexts	Class	19
Contexts	Casino	18
Contexts	Stress	15
Contexts	Weight Control	1
Policy	Ads Seen	336
Policy	Warnings Impactful	219
Policy	Smoking Ban	171
Policy	Warnings Not Impactful	130
Policy	FDA	100
Policy	E Cig Ban	72
Policy	Tob Regulation	69
Policy	Taxes	39
Policy	Lung Cancer Screening	28
Policy	Price	12
Products	E-Cig	558
Products	Chew	125
Products	Cigars	89
Products	Strips/Orbs	70
Products	Snus	60
Products	Additives	37
Products	Brand Loyalty	26
Products	Menthol	25
Products	Nicotine	20

When analyzing themes across segments on a particular topic, we sorted rows by code to create collections of segments by topic of discussion. Finally, in order to be sure we included all relevant segments in a collection, we searched for specific key terms (e.g. nicotine, vape, ban, etc.) across the focus group transcripts and in this way updated the coding until we were satisfied that transcripts had been adequately coded. Finally, we were able to refine our collections by sorting the spreadsheet by combinations of codes, e.g. “nostalgia” and “ads effective”. The present paper focused on segments coded as related to messages, warnings impactful, warnings not impactful, smoking bans, and tobacco regulation.

## Results

The sample included, 51 current and former smokers, ages 45 to 68 (mean age 52.6; SD 6.1) (Table 2). Generally, warning labels and anti-smoking warnings were seen as either ineffective or counter-productive for smoking cessation, and several types of anti-smoking messages were identified as more salient than others.

**Table 2:** **Participant demographic characteristics (N = 52)**

**Characteristic**		**Number (%)**
Age		
	45-49	20 (38.5)
	50-59	24 (46.1)
	60+	8 (15.4)
Female		15 (29)
Race/ethnicity		
	White, non-hispanic	26 (50)
	Black, non-hispanic	17 (33)
	Other, non-hispanic	8 (15)
	Hispanic	1 (2)
Education		
	Some college or higher	43 (83)
Annual Income (thousand USD)		
	<40	22 (42)
	40-100	27 (52)
	>100	3 (6)

### Anti-smoking warnings as a smoking Cue for older smokers

None of the older smokers in these focus groups deemed anti-smoking warnings effective for promoting cessation. Some even felt that the anti-smoking “ads” were counter-productive because they triggered urges for a cigarette.

You know, a few years ago, they used to have a lot of billboards that just plainly say ‘stop smoking’. I hated those ads, because, you know, I wouldn’t even have thought of a cigarette, and then I would be driving, and I see that sign. Boy, I’m instantly thinking about having a cigarette. I used to hate those billboards.

Another participant commented:

I can give you a bad story. . . When [they] first came out, I had quit. Before I went to the store, I saw one of these antismoking ads, and it reminded me about smoking. I went and bought cigarettes . . . they show people smoking and talking about it, and that got me. I [had] quit for like three or four weeks . . .

### Older smokers ignore warnings on cigarette packages

Participants described cigarette warnings as easy to ignore, especially small text messages printed on cigarette packages. “There’s definitely warnings on cigarette packs, but I don’t look at them.” Another participant elaborated, “Its sort of like brussel sprouts, you ignore them. . . You write them off because you’re not interested in them”.

CDC graphic warning labels were shown to all participants, and they were asked to discuss them in terms of relative rating of impact and effectiveness for the promotion of smoking cessation. Photographs of cancerous lung and oral tissue and smoke coming out of a tracheostomy were identified as the images with the most “impact’. However, none of the nine images (Figure 1) were seen as an effective motivator to quit smoking. Referring to even more graphic warnings seen while traveling outside the US, one participant remarked, “We’ve looked *through* that . . . If you’re a smoker, you just look past it”. When the US eventually requires larger, more graphic warnings on cigarette packs, this participant has a solution: “I got a business already figured out. . . You put the cigarettes in the case and it just blocks the photo—the bottom half”.

### Televised anti-smoking messages noticed but not effective

Older smokers are able to compare current messages with anti-smoking messages from the past and agree that images and messages have become more intense but not necessarily more relevant. “I mean, there's always been people who have been severely debilitated by cigarette smoking, but, like, we haven’t always seen them in the way that they are on now”. Another participant described how repeated exposure to anti-tobacco messages simply conditioned them to develop a higher tolerance that left them inured to the perceived escalation of warnings.

I mean, I don't take it personally. No. I mean. . . there’s been antismoking advertising for a long time, but it feels like they’ve really come to this point where it’s like, okay, no, now we’re really serious, a. . . look at this. This is like -- you know, this is horrible. . . you know what I’m saying?

For some, anti-smoking messages trigger feelings of shame for continuing to smoke despite knowledge of the risks, a type of reaction that fits a self-blaming narrative common among older smokers.

I usually feel like I don’t want to look, and I guess I feel kind of embarrassed in a weird way because I still smoke. . . Like, look how horrifying this is. And, yeah, it’s something that I’m still doing.

Participants offered examples of rationalization common among smokers, they discussed people who have lived a long life despite smoking, thereby neutralizing the impact of the message.

My grandmother lived to 80 and smoked right up until the end. Never had anything. So out of the antismoking commercials and all that, I go, “What about my grandmother?” I mean, it’s almost like a crapshoot as much as it is smoking.

Participants suggested that while “grotesque” anti-smoking ads may grab the attention of older smokers, the message of the ad was not readily recalled.

I saw an ad today . . . that freaked me out. I mean, it was really grotesque, but I don’t remember any of the details about it, but I know it caught my attention. . . It was the most over-the-top one I’ve ever seen on TV. I can’t give you any of the details about it because it just freaked me out, and I’m like, come on. You’re going too far.

The televised warning referred to as “the lady with the hole in her neck” was mentioned more than any other ad. The ad had impressed the participants with its overall “advertising quality”, and its alarming content was emphasized.

When asked by the moderator, “[could it] change your thoughts about whether you wanted to quit or not…?“one participant responded “No. It just felt disturbing …as it would be disturbing to look at people clubbing seals”. This anti-smoking message, although memorable and disturbing, had not motivated any of them to quit smoking. Some interpreted the ad as a testimonial to the power of nicotine addiction. As noted by one, “. . . ask them people with a hole in their throat. Actually, it doesn’t affect them enough to make them quit smoking”. Another conceded, “Oh, when you see that, you don’t want to have a cigarette *at that moment”.*

### Relevant and engaging messages for older adults

General themes and content of anti-smoking messages and warning labels were discussed. Four categories were identified as the most compelling.

#### Child losing a parent

Warning labels and anti-smoking messages related to a child losing a parent to tobacco-related disease resonated strongly with participants.

You know, my girlfriend who had smoked, she started so young. And the one thing that got her to quit, which, unfortunately, I don’t have, is her daughter said, ‘Mom, I don’t want you to die. I want you to live. And I want you to still be around and be my Mommy’. I don’t have any kids. I think if a kid said that to me, that would probably make me stop.

#### Second hand smoke

When asked by the moderator if messages about second hand smoke had influenced their smoking behaviors, most participants let out a chorus of “No”, and “Not me”. However, one remarked “Actually, I do think about second-hand smoke now more than I ever did. . . I understand (the girl that works in the bar) doesn’t have to smoke cigarettes—inhale tobacco smoke all her eight hour shift. Now, I understand that. So, these ads have changed my behavior”. The focus of second hand smoke on children was identified as the most powerful; the example given was, “she’s smoking on the balcony and the smoke drifts up and kills the baby”.

#### Real-time tabulation of smoking-related deaths

One participant favored another type of warning. “I was just thinking of an electronic billboard they have in Westwood on the drive by Santa Monica on the 405 Freeway . . . It says – it’s a counter that goes up every few seconds to say, ‘This is how many people die every day from cigarettes’. And that’s like another good one. I like that one”. Others agreed, although, they also agreed that the message had not affected their behavior.

#### Positively framed messages

Some participants suggested that anti-smoking ads that have a positive or pro-active focus may be more effective than negative warnings. Many expressed an interest in messages that focus on the benefits of cessation not the risks of smoking.

The negative commercials are less effective than the more positive ones. They had one California put out which was bubbles. It had bubbles in it, and it was people blowing bubbles instead of smoking cigarettes, and that . . . got to me on a much better level. . . The whole—the only anti-smoking stuff you see is negative. It’s all negative, and it’s ugly, and it’s difficult to look at. I think it might be very interesting if they just came out and said, ‘If you’re 40 years old and are a smoker and you quit smoking today, you could add ten years to your life. . . . Positive things, positive. . . . Acknowledge the fact you’re a smoker and why should you quit. This is why you should quit.

## Discussion

The findings of this study suggest that urban and suburban older smokers participating in our California focus groups do not believe that current warning labels and anti-tobacco messages are effective for the promotion of cessation among long-term smokers. Anti-tobacco messages recalled by study participants had not motivated them to quit smoking, and warning labels on cigarette packages were deemed equally ineffective. The participants emphasized how easy it is to ignore negative messages, and some noted that anti-smoking messages can trigger cravings for a cigarette. Given that the prevalence of tobacco use in older adults is not decreasing at the same rates as other age groups and that the tobacco companies continue to target older smokers with conventional and alternative product advertisements, these findings suggest that anti-tobacco messages specifically targeted to older smokers are urgently needed.

The results from this study demonstrate the importance of further investigation of how age cohort affects perceptions of anti-tobacco messages, particularly regarding the effectiveness of these messages as motivation for smoking cessation. Unlike younger adults, older smokers came of age when smoking was ubiquitous, and cigarettes were advertised on television, radio, and billboards. As a result, older smokers may respond to anti-smoking messages and cigarette package warning labels differently than younger smokers. Most older smokers were already smoking at the advent of warning labels. This cohort effect may be a significant influence on long-term smoker receptivity to anti-smoking messages.

The literature on whether anti-smoking messages can provide smoking cues and increase urges is contradictory. In one study, a trend was found toward greater increases in smoking urges following viewing of anti-tobacco messages with smoking cues [32]. Another study suggested that smoking cues may undermine the persuasive effects of anti-smoking messages in former smokers, although there was no effect on smoking urges [33].

The results of our study support the premise that when anti-smoking messages include smoking-related cues in order to illustrate the negative consequences of smoking they can trigger cravings to smoke and may play a role in relapse [34,35]. Our findings contradict a more recent study by Falcone et al. with 319 participants (age range 20–61, mean age 32.5, SD 9.9) that found that visual smoking cues in anti-tobacco messages do not increase urges to smoke [36]. However, all of these studies differ by demographics and study designs that may account for differing results. Most studies did not find a significant effect from age, but that may be because of the lack of variance in the samples (i.e., too few older smokers).

The argument has been made that the variable in the equation is whether the message is strong enough to outweigh the potential trigger for a craving. Prior evidence suggests that smoking cues in anti-tobacco public service announcements can increase smoking urges if the central argument is weak [32]. Given that older smokers have been exposed over time to thousands of anti-tobacco messages, the strength of the messages may be eroded. Furthermore, smokers display attentional biases to smoking cues that may affect cognitive processing of the message [37,38]. By distracting smokers from the central message and providing a clear motivator to continue smoking (i.e., increased urge to smoke), the presence of smoking cues in anti-smoking messages could be counter-productive to the goal of reducing smoking prevalence for older adults.

Older adults are knowledgeable about the risks and health effects of smoking, however they tend to be less knowledgeable about the benefits of cessation and may underestimate their ability to quit. Combined with the preference for positively framed messages, more messages are needed that outline the immediate as well as the long-term benefits of cessation for older smokers. These messages could be presented with self-affirming images such as multi-generation activities (i.e., older adults interacting with children in a healthy smokeless environment).

Our study supports previous studies that found text-only warning labels ineffective, possibly due to desensitizing effects of repeated exposure, which is more salient among older smokers than younger smokers [22,23]. In addition, one participant highlighted the problem of consistent placement of text or graphic messages on the package by suggesting the development of cigarette cases that could cover the warnings.

A strength of this study is that qualitative data provide rich textual descriptions of older smoker’s complex perceptions and reactions to warning labels and messages. This information is needed to tailor anti-smoking campaigns that are relevant to long-term smokers. On the other hand, a limitation of this study is that findings cannot be generalized to other populations or used to make causal claims about the relationship between perceptions of warnings and smoking behavior. Future studies are needed to identify: the types of messages most salient to older adults; how best to deliver the messages; and how anti-smoking messages are mediated by the biographical and social contexts of older adults. Further research is needed on the perceptions of older smokers on anti-tobacco messages. Quantitative national studies are needed. Given the findings of this study and the 19.5% prevalence rate of smoking in the 45–64 age group, anti-tobacco messages designed to resonate with older smokers are needed. Moreover, as the world’s population ages, including older smokers in all aspects of tobacco control and research is imperative. Stepping up efforts to understand the response of older smokers to anti-tobacco messaging can help mitigate the efforts of the tobacco industry to keep older smokers smoking.
